# A Scalable Radiomics- and Natural Language Processing–Based Machine Learning Pipeline to Distinguish Between Painful and Painless Thoracic Spinal Bone Metastases: Retrospective Algorithm Development and Validation Study

**DOI:** 10.2196/44779

**Published:** 2023-05-22

**Authors:** Hossein Naseri, Sonia Skamene, Marwan Tolba, Mame Daro Faye, Paul Ramia, Julia Khriguian, Marc David, John Kildea

**Affiliations:** 1 Medical Physics Unit McGill University Health Centre Montreal, QC Canada; 2 Division of Radiation Oncology McGill University Health Centre Montreal, QC Canada

**Keywords:** cancer, pain, palliative care, radiotherapy, bone metastases, radiomics, natural language processing, machine learning, artificial intelligent, radiation therapy

## Abstract

**Background:**

The identification of objective pain biomarkers can contribute to an improved understanding of pain, as well as its prognosis and better management. Hence, it has the potential to improve the quality of life of patients with cancer. Artificial intelligence can aid in the extraction of objective pain biomarkers for patients with cancer with bone metastases (BMs).

**Objective:**

This study aimed to develop and evaluate a scalable natural language processing (NLP)– and radiomics-based machine learning pipeline to differentiate between painless and painful BM lesions in simulation computed tomography (CT) images using imaging features (biomarkers) extracted from lesion center point–based regions of interest (ROIs).

**Methods:**

Patients treated at our comprehensive cancer center who received palliative radiotherapy for thoracic spine BM between January 2016 and September 2019 were included in this retrospective study. Physician-reported pain scores were extracted automatically from radiation oncology consultation notes using an NLP pipeline. BM center points were manually pinpointed on CT images by radiation oncologists. Nested ROIs with various diameters were automatically delineated around these expert-identified BM center points, and radiomics features were extracted from each ROI. Synthetic Minority Oversampling Technique resampling, the Least Absolute Shrinkage And Selection Operator feature selection method, and various machine learning classifiers were evaluated using precision, recall, *F*_1_-score, and area under the receiver operating characteristic curve.

**Results:**

Radiation therapy consultation notes and simulation CT images of 176 patients (mean age 66, SD 14 years; 95 males) with thoracic spine BM were included in this study. After BM center point identification, 107 radiomics features were extracted from each spherical ROI using pyradiomics. Data were divided into 70% and 30% training and hold-out test sets, respectively. In the test set, the accuracy, sensitivity, specificity, and area under the receiver operating characteristic curve of our best performing model (neural network classifier on an ensemble ROI) were 0.82 (132/163), 0.59 (16/27), 0.85 (116/136), and 0.83, respectively.

**Conclusions:**

Our NLP- and radiomics-based machine learning pipeline was successful in differentiating between painful and painless BM lesions. It is intrinsically scalable by using NLP to extract pain scores from clinical notes and by requiring only center points to identify BM lesions in CT images.

## Introduction

### Overview

Most patients with cancer with bone metastasis (BM) experience pain [1] and most receive radiotherapy to control it [2]. But, it has been shown that due to the subjective and qualitative nature of the pain, clinicians often underestimate pain [3]. As a result, many patients with BM receive radiotherapy after their pain has already become debilitating [4].

Although patient-reported outcomes can be used to obtain pain scores directly from patients themselves, the efficacy of these pain scores is limited due to the fact that these ratings are highly qualitative and subjective [5]. Because of this, it is desirable to have pain scoring systems that are more objective. The goal of this study was to explore ways to automatically and objectively quantify pain associated with BMs using computed tomography (CT) images.

We hypothesized that tumor features extracted from CT images of BMs contain imaging biomarkers that may be used to objectively identify BM-associated pain. These pain biomarkers may provide the opportunity to develop objective pain scoring tools to aid in the diagnosis, treatment, understanding, and prognosis of BM pain.

### Background

The search for imaging and nonimaging pain biomarkers has been the focus of numerous studies [5-12]. Various studies [13-21] have shown how artificial intelligence (AI), including machine learning and radiomics, can be used to understand and quantify pain. For example, Mashayekhi et al [22] showed that radiomic features extracted from the CT images of the pancreas can help to identify functional abdominal pain in patients. Vedantam et al [23] explored the viability of using radiomics features extracted from magnetic resonance images to detect pain following percutaneous cordotomy. At least 1 study [13] has reported using radiomics to identify painful metastatic lesions in radiographic images. However, we found no reports in the literature of a scalable approach that can be used efficiently on a large set of unlabeled patient data. To the best of our knowledge, our work is the first to combine natural language processing (NLP) and radiomics to enable an efficient and scalable pain identification pipeline using unstructured data.

A fundamental challenge in developing any AI model for use in medicine is the need to obtain sufficient patient data for training and testing. For example, the data set used by Wakabayashi et al in the study that we mentioned earlier [13], was limited to 69 patients. One limiting factor is obtaining standard patient-reported pain scores for use as ground-truth data, and another limiting factor is obtaining segmented images from which to extract tumor biomarkers. For the work reported in this paper, we overcame the data set size limitation by using 2 novel strategies. First, by combining NLP with radiomics, we quickly mined pain scores from clinical notes and used these NLP-extracted scores to label our radiomics features for supervised learning. Second, by asking our clinical colleagues to pinpoint only the center points of BM lesions in radiotherapy simulation CT images, we maximized the number of lesions identified in the time available. In the medical field, NLP has shown promising results in extracting biomedical information and clinical outcomes such as pain from unstructured text data [24-26]. Moreover, as we reported previously [21], by automatically delineating geometrical regions around BM lesion center points, it is possible to successfully extract radiomics features for robust BM lesion detection. In this study, we report how our combined radiomics-NLP machine learning pipeline can successfully identify pain in radiotherapy simulation CT images of patients with cancer with BMs.

## Methods

### Ethical Considerations

This retrospective study was approved by the research ethics board of the McGill University Health Centre (2020-5899) with the waiver of informed consent. We confirm that the entire research was performed in accordance with research ethics board’s guidelines and regulations.

### Data Selection

**Figure 1:**
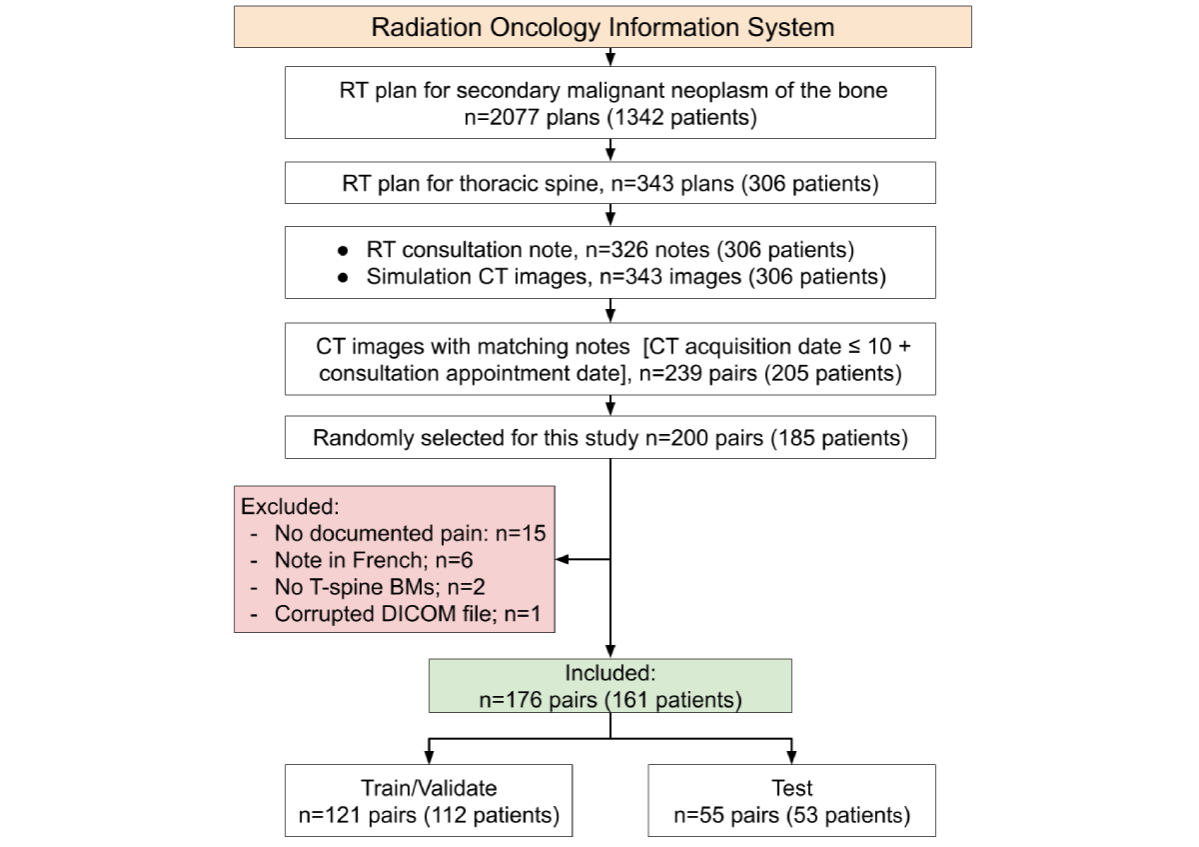
The patient selection criteria used to obtain the radiotherapy consultation notes and simulation computed tomography (CT) images that formed our training and test data sets. The initial number of 200 note-image pairs included in this study was based on the minimum sample size calculation as explained in Section A.1 in Multimedia Appendix 1. BM: bone metastases; DICOM: Digital Imaging and Communications in Medicine; RT: radiotherapy; T-spine: thoracic spine. *Four patients had pairs in both the training and test sets.

Our patient-selection process is outlined in Figure 1. The initial number of 200 pairs of radiation oncology consultation notes and CT images of patients with spinal BM were included in this study based on the minimum sample size calculation as explained in Section A.1 in Multimedia Appendix 1 [27]. In total, 120 of the notes and all 200 of the CT images from this study were independently used in 2 studies we previously reported on [21-25]. The first [25] of these studies showed the feasibility of extracting pain from consultation notes of patients with cancer, using NLP. The second [21] demonstrated the feasibility of using lesion center point–based radiomics models to differentiate healthy and metastatic bone lesions in CT scans of patients with BMs. This study combined the data and results from these 2 prior studies and expanded upon them to build an NLP- and radiomics-based model to detect pain using the CT scans of patients.

We searched our institution’s Oncology Information System for the radiotherapy plans of patients diagnosed with a “secondary malignant neoplasm of bone” between January 2016 and September 2019. From the retrieved list, we selected those who were treated for thoracic spinal BM. Then, we retrieved the corresponding consultation notes and simulation CT images. A note-image pair was included if (1) the note was in English, (2) pain was documented, (3) the simulation CT image was taken up to 10 days post consultation, and (4) simulation CT revealed BM lesions in the thoracic spine. Patients with multiple but nonoverlapping note-image pairs were considered independent samples. We only considered the same patients as new participants if they had CT scans and associated consultation notes for BM lesions in different areas of their spines. As a result, each BM lesion was included only once in our study. Also, it should be noted that palliative patients normally have their simulation CT scan (for treatment planning) on the same day or within a few days after the consultation, and radiotherapy is delivered on the same day or within a few days after treatment planning. To assure that there is no change in the BM lesion structure or pain status, we did not allow more than a 10-day gap between the two. Figure A1 in Multimedia Appendix 1 displays the distribution of the time interval between the radiotherapy consultation and CT acquisition dates.

We randomly assigned note-image pairs to the training or cross-validation set (approximately 70%) or the holdout test set (approximately 30%). We used stratified randomization to preserve the original sample ratio between pain labels in each sample set. In addition, we performed a paired *t* test and a chi-square analysis [28] to ensure that there was no systematic bias in any of our sample sets regarding gender, age, or primary cancer type. Patient demographics are presented in Table 1.

**Table 1:** Patient demographics in the training and test sets.

Characteristics		Training and validation set (n=121)	Test set (n=55)	*P* value^a^	
**Gender, n (%)**					N/A^b^
	Female	56 (46)	25 (45)		
	Male	65 (54)	30 (55)		
**Age (years), mean (SD)^c^**					N/A
	Female	63 (14)	64 (12)	.99	
	Male	67 (14)	64 (13)	.72	
**Primary cancer type, n (%)**					.06
	Lung	32 (26)	20 (36)		
	Breast	23 (19)	11 (20)		
	Prostate	19 (16)	5 (9)		
	Multiple myeloma	8 (7)	6 (11)		
	Renal cell carcinoma	7 (6)	2 (4)		
	Other and unknown	64 (53)	31 (56)		
**Bone metastasis lesions, n (%)**					.42
	Lytic	220 (52)	76 (47)		
	Blastic	122 (29)	57 (35)		
	Mix	81 (19)	30 (18)		
**Pain label, n (%)**					N/A
	Pain	357 (84)	136 (83)		
	No pain	66 (16)	27 (17)		

^a^*P* values for numerical values (age) and categorical features (primary cancer site and bone metastasis lesion type) were calculated using a 2-tailed heteroscedastic *t* test and a chi-square test, respectively.

^b^N/A: not applicable.

^c^The *P* value for the age difference between males and females was .20 for the training and validation set and .50 for the test set.

### NLP-Extracted Pain Labels

Due to the absence of patient-reported pain scores in our Oncology Information System, we extracted physician-reported pain scores from patients' radiation oncology consultation notes using our previously reported NLP pipeline [25]. While pain scores were typically reported as part of the “history of the present illness” in our hospital, for the sake of generalizability, we extracted pain scores from the entire note.

Our NLP pipeline first processed the text with MetaMap [29] and mapped it to the UMLS (ie, Unified Medical Language System) Metathesaurus [30] in order to identify pain terminologies and their severity scores. Next, it applied rules to filter out hypothetical, conditional, and historical references to pain in order to focus solely on references to pain at the time of the consultation. Then, it calculated the average pain intensity (API) in each note by averaging the pain scores therein. Finally, it assigned each note a “verbally declared pain” (VDP) label, as VDP=“no pain” (if API 0), and VDP=“pain” (if API0). These pain labels were used to train, validate, and test our radiomics model.

### Expert-Extracted Pain Scores

To evaluate the effect of NLP-extracted pain labels on the performance of our pipeline, we also generated best-available ground-truth pain labels using expert-annotated pain scores. To do so, our radiation oncologists used the texTRACTOR [31] pain labeling application to manually read consultation notes and label valid pain scores in our training and test data sets using a 4-grade verbal rating scale (no pain, mild, moderate, and severe). A mention of pain was regarded as valid if it reflected the status of pain at the metastatic sites for which treatment was planned at the time of the consultation. Table A1 in Multimedia Appendix 1 contains all the NLP- and expert-extracted pain scores, and Figure A2 in Multimedia Appendix 1 illustrates the level of agreement between them. Due to the quality of the documented pain scores and lack of interrater agreement among experts (Fleiss κ=0.43), as explained by Naseri et al [25], we subsequently defined a binary pain score as “no pain” and “pain” in order to establish satisfactory interrater agreement (κ=0.66) [25]. To create binary ground-truth pain labels comparable to the NLP-extracted labels, we assigned notes scored as “no pain” to “no pain” and notes scored as “mild,” “moderate,” and “severe” pain to “pain.” These expert-extracted pain scores were used to measure how well the NLP pipeline works.

### Center Point Identification of BM Lesions

BM lesion center points were identified by a team comprising a staff radiation oncologist (SS) with 10 years’ experience, a radiation oncology fellow (MT), and 3 third-year radiation oncology residents (J Khriguian, PR, and MF). Simulation CT DICOM (ie, Digital Imaging and Communications in Medicine) files were exported from the radiotherapy treatment planning software and deidentified. Then, the CT images were randomly divided into 5 sets and loaded into the diCOMBINE [32] application for BM lesion center point identification. Our experts were blinded to patients’ pain statuses and identities. We requested each expert to label center points for all visually identifiable BM lesions in all CT images within 1 of the 5 sets, and another expert was assigned to validate their labels. A key benefit of this radiomics pipeline [21] is that it does not require full lesion segmentation, making it feasible to engage busy clinicians.

### Segmentation of Regions of Interest

Using our previously reported methodology [21], we automatically segmented lesion center point–based nested spherical (SP) regions of interest (ROIs). To do this, we first delineated nested spherical ROIs around the identified BM lesion center points (see Textbox 1, top panel). ROI diameters ranged from 7 mm (3×3 voxels) to 50 mm (average size of the vertebral body) [33]. Then, in addition to what was reported by Naseri et al [21], we used Hounsfield units thresholding to exclude fat and air regions from the delineated ROIs. For this, motivated by Deglint et al [34] and Ulano et al [35], we applied a threshold to remove voxels with negative Hounsfield units from our ROIs. Hounsfield units of <0 are associated with fat and air [34]. We used OpenCV [36] (version 4.4.0) for Hounsfield units thresholding and applied a Gaussian filter to reduce noise. Then, we used pynrrd [37] (version 0.4.2) to export each ROI as a 3D binary mask and store it as a.nrrd [38] file. Finally, we aggregated these nested ROI masks to form ensemble ROIs. In this study, we examined 2 contrasting ensemble (EN) ROIs as shown in Textbox 1 (bottom panel): one with small size and 3 layers (EN3) and the other with large size and 6 layers (EN6). Wakabayashi et al [13] and Naseri et al [21] have shown that radiomics-based machine learning models trained on ensemble ROIs have better classification performance than single ROI–based models.

**The characteristics of the spherical and ensemble regions of interest (ROIs) used in this study.**

Nested spherical (SP) ROIs with Hounsfield units (HUs) intensity thresholds (HU>0):

- SP7 (diameter 7 mm)
- SP10 (diameter 10 mm)
- SP15 (diameter 15 mm)
- SP20 (diameter 20 mm)
- SP30 (diameter 30 mm)
- SP50 (diameter 50 mm)

Ensemble (EN) ROIs:

- EN3 (ROI SP7+SP10+SP15)
- EN6 (ROI SP7+SP10+SP15+SP20+SP30+SP50)

### Radiomics Models

**Figure 2:**
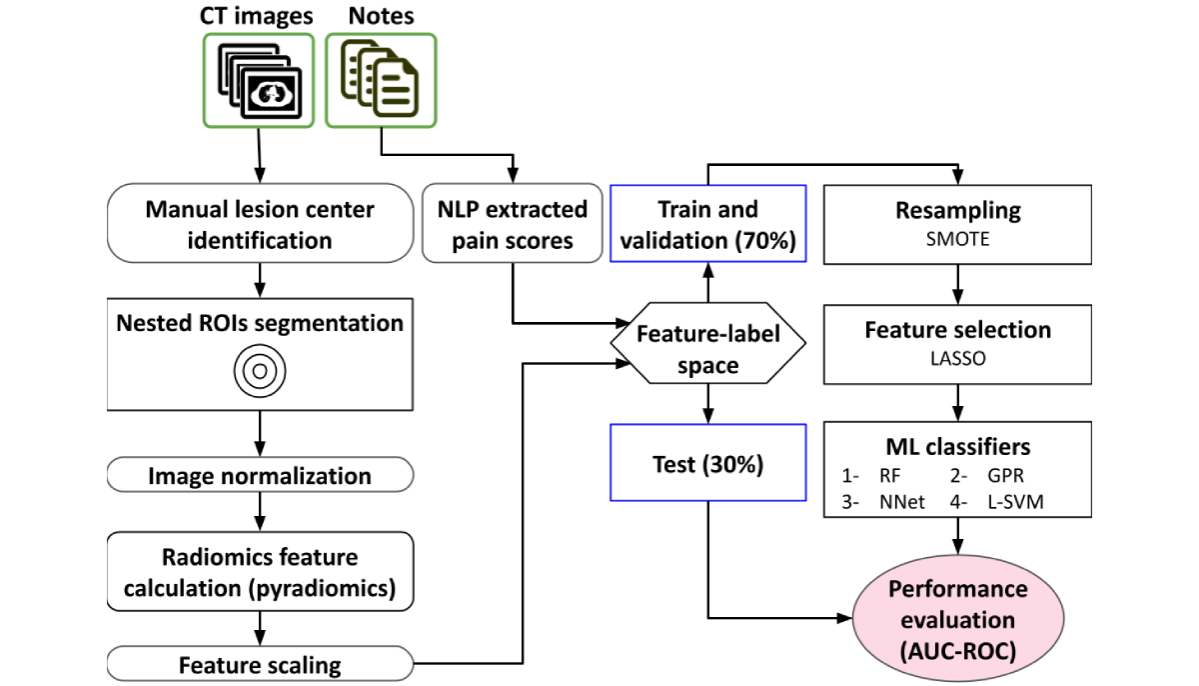
The radiomics-based pipeline that we used to select and train a machine learning model to separate painful and painless bone metastasis lesions. Our pipeline is the same as that published by Naseri et al [21] but using NLP-extracted pain labels and modified to account for sample imbalance. AUC-ROC: area under the receiver operating characteristic curve-receiver operating characteristic; CT: computed tomography; GPR: Gaussian process regression; LASSO: Least Absolute Shrinkage And Selection Operator; L-SVM: linear support vector machine; ML: machine learning; NLP: natural language processing; NNet: neural network; RF: random forest; ROI: region of interest; SMOTE: Synthetic Minority Oversampling Technique.

Our radiomics pipeline is illustrated in Figure 2. We essentially used our previously reported pipeline [21] but with our NLP- and expert-extracted pain labels to train and test it. We made one improvement to the pipeline by incorporating Imbalanced-learn [39] (version 0.7.0) as a resampling step to account for imbalance (see below).

Radiomics features were extracted from each CT image using masks composed of the ensemble ROIs listed in Textbox 1. Then, the feature space was scaled using *z* score normalization [40], and the associated NLP-extracted binary pain labels (pain=1, no pain=0) were incorporated. A single NLP-extracted pain score was assigned to all the lesions extracted from a given paired CT image.

Due to the nature of BM pain [41], there was a large imbalance between the number of painful and painless lesions (493 pain, 93 no pain). Therefore, we used the Synthetic Minority Oversampling Technique (SMOTE) [42] in the training phase as it has been shown to be the best-performing resampling method for radiomics [43]. We did not apply resampling to our test set in order to maintain the original sample imbalance. Then, the Least Absolute Shrinkage And Selection Operator [44] feature selection method was applied to the feature space to remove noninformative features. Least Absolute Shrinkage And Selection Operator is a commonly used feature selection method in radiomics studies [45,46]. Finally, we examined the Gaussian process regression, linear support vector machine, random forest, and neural networks classifiers, as they were the best performing machine learning classifiers in our previous work. We evaluated the performance of our models on the training set using 5-fold cross-validation. Final evaluation was performed on the test set. The receiver operating characteristic (ROC) [47] curve, area under the ROC curve (AUC), precision, sensitivity, specificity, and *F*_1_-score metrics were used to report the performance of our models on the training and test sets. We also trained and tested our best performing pipeline using the expert-extracted pain scores (best-available ground-truth) to evaluate the impact of NLP-extracted pain labels.

## Results

### Patient Demographics

A total of 176 pairs of radiotherapy consultation notes and simulation CT images of patients with thoracic spinal BM were included in this study. As summarized in Table 1, a total of 121 sample pairs (mean patient age 63, SD 14 years; males: n=65, mean age 67, SD 14 years; *P*=.20) were included for training and cross-validation, and 55 sample pairs (mean patient age 64, SD 12 years; males: n=25, mean age 64, SD 13 years; females: mean age 64, SD 23 years; *P*=.50) were included in the test set. The sample selection procedure and data quantities are presented in Figure 1. The demographics of the patients in the training and test sets are presented in Table 1. The most common primary cancer sites were the lungs (n=52), breasts (n=34), and prostate (n=24).

A total of 586 BM center points were identified by our experts on the training (n=423 lesions) and test (n=163 lesions) data sets. In the training set, 357 (84%) lesions were labeled by the NLP pipeline as painful and 66 lesions were labeled as painless. In the test set, 136 (83%) lesions were identified by the NLP pipeline as painful, and 27 lesions were labeled as painless. This represented a significant but equal imbalance in our training and test sets.

### Segmented ROIs

**Figure 3:**
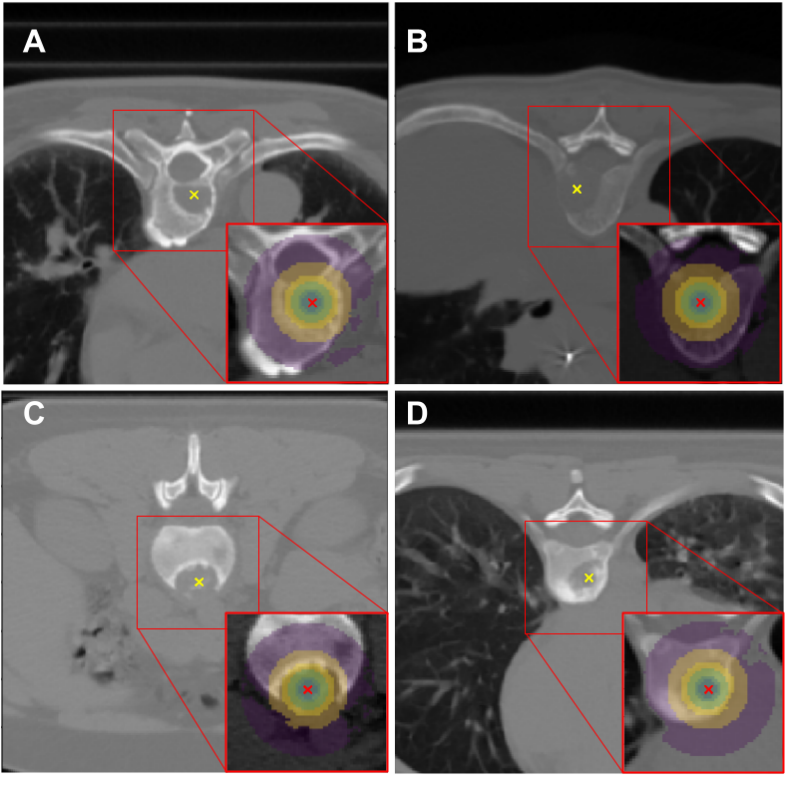
Examples of segmented nested spherical regions of interest (ROIs) with the Hounsfield units threshold applied on computed tomography images of patients with painful (A, B) and painless (C, D) bone metastases lesions. Nested ROIs with diameters of 50, 30, 20, 15, 10, and 7 mm are shown in the insets as different hues.

Examples of segmented ROIs with the Hounsfield units threshold applied are presented in Figure 3 for painful and painless BMs.

### Testing Our Radiomics Models

**Figure 4:**
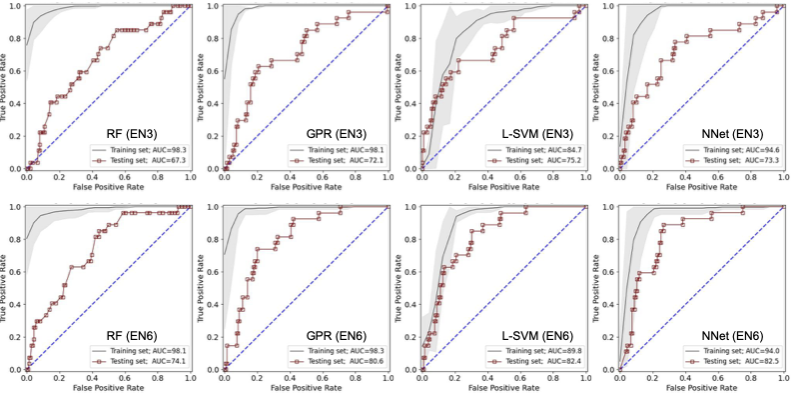
Receiver operating characteristic curves for our classifiers using 3-layer ensemble (EN3) (top row) and 6-layer ensemble (EN6) (bottom row) lesion center point–based ensemble regions of interest in training (black lines) and test (dark red squares) data sets. AUC: area under the receiver operating characteristic curve; GPR: Gaussian process regression; L-SVM: linear support vector machine; NNet: neural network; RF: random forest.

In total, 107 radiomics features were extracted from each of the 6 nested ROIs. Then, they were aggregated to form feature spaces for the EN3 (with 321 features) and EN6 (with 642 features) ensemble ROIs. Figure 4 shows the ROC curve of each model in the training (black lines) and test (red squares) data sets using the EN3 and EN6 ROIs. On the training set, the gray range represents the mean (SD) AUC of the 5-fold cross-validation. The AUC and *F*_1_-score grids are presented in Table 2.

The precision, accuracy, sensitivity, specificity, *F*_1_-score, and AUC values of our best-performing pipeline (neural networks with the EN6 ROI) are presented in Table 3. The performance of this pipeline (trained and tested) on the data set of expert-extracted pain labels (best-available ground-truth) is provided as a quality measurement. The performance of the model described previously by Wakabayashi et al [13] is also provided for comparison.

**Table 2:** The area under the receiver operating characteristic curves (AUCs) and *F*_1_-scores of our machine learning classifiers in the training and test data sets using the ensemble (EN) regions of interest EN3 and EN6 for each of the RF (random forest), GPR (Gaussian process regression), L-SVM (linear support vector machine), and NNet (neural networks) classifiers.

Region of interest		Training set					Test set			
		RF	GPR	L-SVM	NNet	RF		GPR	L-SVM	NNet
**Areas under the receiver operating characteristic curve**										
	EN3	98.3	98.1	84.7	94.6	67.3		72.1	75.2	73.3
	EN6	98.1	98.3	89.8	94.0	74.1		80.6	82.4	82.5
***F*_1_-scores**										
	EN3	90.0	89.9	79.4	90.5	60.9		64.7	65.4	63.6
	EN6	93.0	93.0	84.7	91.6	63.8		66.9	67.4	69.5

**Table 3:** The performance of our best-performing natural language processing (NLP)–radiomics pipeline (neural networks with the ensemble 6 region of interest) on the training and test sets using NLP and manually extracted pain labels, together with the results from a prior study by Wakabayashi et al [13].

	Accuracy	Precision	Sensitivity	Specificity	*F*_1_-score	AUC^a^
This study (training set)	92.4	93.2	92.4	86.4	91.6	94.0
This study (test set)	81.0	67.9	59.2	85.3	69.5	82.5
This study (training set); using manual pain scores	94.2	94.8	98.7	89.7	94.4	98.1
This study (test set); using manual pain scores	83.5	64.9	64.7	85.7	68.0	82.3
Wakabayashi et al [13] (training test only)	73.9	—^b^	71.0	86.0	—	82.0

^a^AUC: area under the receiver operating characteristic curve.

^b^Not determined.

## Discussion

Underestimation and undertreatment of cancer pain can significantly diminish the quality of life of patients with cancer. Accordingly, systems that can objectively measure cancer pain have the potential to improve quality of life. In this study, we created a scalable NLP-radiomics pain identification pipeline. Our pipeline is designed for palliative treatment for patients with cancer undergoing radiotherapy therapy, for whom there are typically just 2 contemporaneous sources of relevant medical information at the time of the treatment: consultation notes and simulation CT images. We used an NLP pipeline to extract physician-reported pain scores from radiotherapy consultation notes. NLP-extracted pain scores are appropriate, when structured patient-reported pain scores are unavailable (as is the case for at least 25% to 35% of all patients with cancer [13,48] and for all patients with cancer receiving palliative care who are treated with radiotherapy at our institution at the time the data were used in this study). Our lesion center point–based spherical ROI delineation method significantly sped up the ROI segmentation procedure, enabling us to rapidly delineate BM center points in 176 images in this study. For comparison, the radiomics pipeline that was developed by Wakabayashi et al [13] required full 3D segmentation of each ROI (69 images).

Due to the unbalanced nature of BM pain, our data set contained significantly fewer “no pain” samples. In order to better train our models, we applied SMOTE resampling to the training set to balance the number of samples with the NLP-extracted “pain” and “no pain” labels. We did not apply any resampling techniques to our test (hold out) set to maintain the original sample imbalance. Therefore, while our training set was balanced, our test set had 5 times more “pain” cases than “no pain” cases (136 pain versus 27 no pain cases). This caused a significant change in the pipeline’s performance between the training and test sets. It has been shown that oversampling improves the overall performance of machine learning models, but the effect is stronger on the training set due to the inclusion of replicated samples in the cross-validation subsets [49]. Moreover, the imbalance in our test set led to high specificity (ability to properly identify pain instances) and low sensitivity (ability to correctly identify no pain cases) in the performance evaluation. For comparison, the sample imbalance reported by Wakabayashi et al [13] was 2:1, resulting in a more balanced relationship between the sensitivity and specificity of their model.

The performance of our pipeline did not improve much when we trained and tested it using expert-extracted pain labels (best-available ground-truth). This might be the case because, in the first experiment, we both trained and tested our pipeline using NLP-extracted pain labels, and in the second experiment, we both trained and tested our pipeline using expert-extracted pain labels. Consequently, after being trained with one set of labels (NLP- or expert-extracted), our pipeline performed well on the test set that was labeled using the same method (NLP or expert). We also demonstrated that our pipeline’s performance is comparable to that of Wakabayashi et al [13], who achieved their results using patient-reported pain labels.

Our pipeline performed significantly better on the EN6 ROIs than on the EN3 ROIs. This could be the case because in comparison to EN3, our EN6 ROIs include additional ROIs with sizes of 20, 30, and 50 mm. From visual inspection, we suspect that, in addition to the characteristics of the BM lesion itself, its location (eg, its proximity to the spinal cord) may be a significant contributor to the BM pain. As a result, larger ROIs enable our algorithm to extract characteristics from outside the BM lesion. Wakabayashi et al [13] also demonstrated the effectiveness of using ROIs outside of the BM lesion.

We are unable to offer a convincing explanation as to why neural networks outperformed random forest and support vector machine classifiers in our analysis. Notwithstanding, it has been demonstrated that neural network classifiers perform better when applied to more difficult problems and larger data sets, while random forest and support vector machine classifiers typically perform well with smaller data sets [46,50,51].

Our pipeline was successful in extracting radiomics biomarkers capable of distinguishing between painful and painless BM lesions. These biomarkers potentially provide the opportunity to objectively identify clinical pain-related indicators that may aid in the diagnosis, treatment, and understanding of BM pain.

Our work has several limitations. First, we used data from a single center for this retrospective study. A multicenter study with a larger data set is necessary to assess the generalizability of our radiomics pipeline for pain quantification. We anticipate that the performance of our NLP-radiomics pipeline will vary based on the pain scoring systems of the cohorts tested. Second, by using lesion center point–based geometrical ROIs, we ignored lesion characteristics such as size and shape, which may be important in the context of pain. Although we used Hounsfield units intensity thresholding to preserve some tumor information, we are considering implementing deep learning–based ROI segmentation in the future as it may better account for full tumor and surrounding tissue characteristics. Lastly, we used SMOTE resampling to address the issue of class imbalance. An alternative solution might be to develop cost-sensitive machine learning classifiers that account for the cost of misclassifying minority samples [52]. However, there is no clear consensus in the literature on whether cost-sensitive learning outperforms resampling [53]. A model that can differentiate between painful and painless lesions from medical imaging is a critical component of any possible radiomics-based pain quantification pipeline. This work not only shows the feasibility of developing a pain quantification tool, but also it removes some of the barriers to its development. As a result, our future work will be to apply our pipeline to patients’ past and current CT images and consultation notes in order to develop a longitudinal model of pain. Such a model should take into account not only images (taken before, during, and after delivering radiotherapy) but also other internal and external parameters that can influence how pain evolves over time (such as primary cancer type, radiation dose, other treatments, and pain medications). Also, it will include patient-reported pain scores to provide more accurate ground-truth pain labels in order to develop a more robust deep learning–based NLP pipeline [24,54]. This, however, is beyond the scope of this investigation.

In conclusion, we demonstrated that our NLP and radiomics-based machine learning pipeline can effectively differentiate between painful and painless BM lesions in simulation CT images using ensemble lesion center point–based geometrical ROIs. Using NLP-extracted pain labels in conjunction with lesion center point–based radiomics features is time efficient. This helps to pave the way for the development of quickly trained and efficient clinical AI-based decision-making tools that can objectively measure cancer pain. Such a tool may help alleviate the burden of pain management and improve the quality of life of patients with BMs.

## Appendix

Multimedia Appendix 1 Sample data.

## Abbreviations

AI: artificial intelligence
API: average pain intensity
AUC: area under the receiver operating characteristic curve
BM: bone metastasis
CT: computed tomography
EN: ensemble
NLP: natural language processing
ROC: receiver operating characteristic
ROI: region of interest
SMOTE: Synthetic Minority Oversampling Technique
SP: spherical
VDP: verbally declared pain
