# Supplementary material for: A Scalable Radiomics- and Natural Language Processing–Based Machine Learning Pipeline to Distinguish Between Painful and Painless Thoracic Spinal Bone Metastases: Retrospective Algorithm Development and Validation Study
Source: JMIR AI. 2023 May 22;2:e44779. doi: 10.2196/44779 (PMC11041487; doi:10.2196/44779)
Supplement: Multimedia Appendix 1 [file ai_v2i1e44779_app1.docx]

## Supplemental Information

### A.1 Sample size calculation

We used Cochran's sample size formula [54] to determine the minimum sample size required to evaluate the performance of the pipeline. An initial audit of our data set showed that the probabilities of finding a patient in our data set with `pain' was 85% (*p_pain_*=0.85) and with ‘no pain' was 15% (*p_no-pain_*=0.15). To ensure that the pain-score detection is precise within a 95% confidence level (*Z_1-α/2_*=1.96), and a 5% margin of error (*e*=0.05), the minimum sample size was determined as,

$$N=p_{pain}*p_{no-pain} (\frac{Z_{1-\alpha/2}}{e})^{2}=0.85*0.15*(\frac{1.96}{0.05})^{2}=196$$

Therefore, we included 200 patients in this study to satisfy the minimum sample size requirement.

### A.2. Time gap between the consultation note and CT acquisition dates


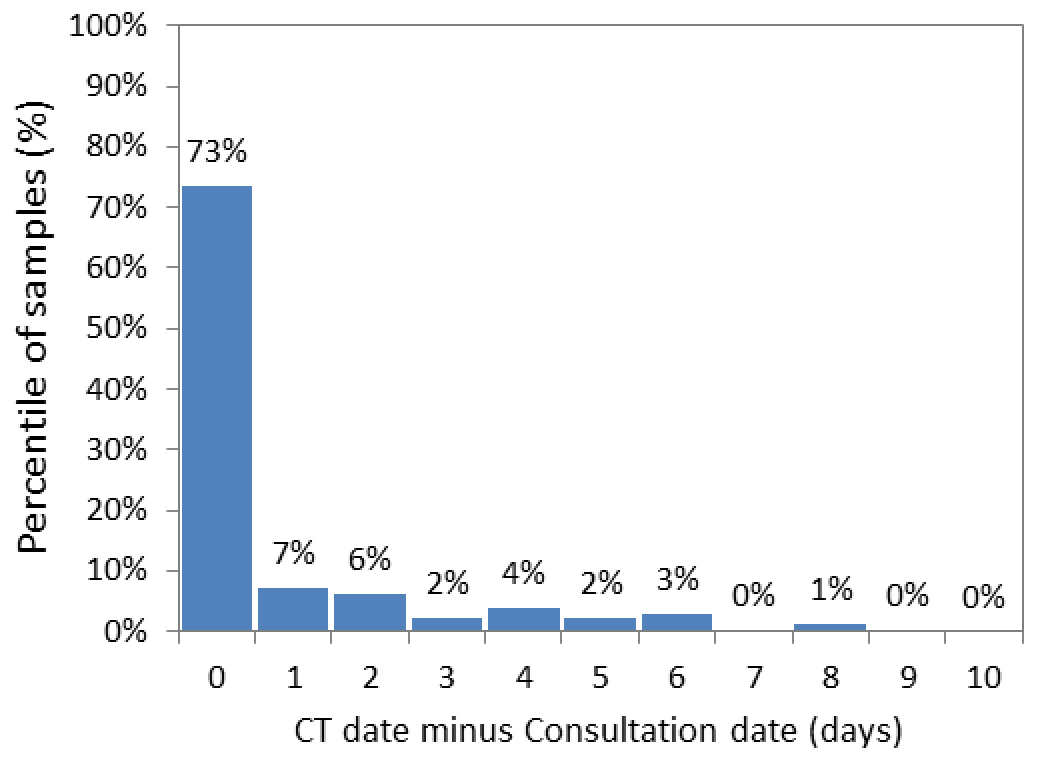


### Figure A1: The distribution of the time interval between the CT acquisition date and RT consultation date (n = 239 pairs).

### A.3. Pain labels

NLP-extracted APIs and VDP values, and expert-extracted pain scores in our database are presented in Table A1. The box plot comparing the distribution of NLP-extracted API values versus expert-extracted pain scores is shown in Figure A2.

**Table A1.** The performance of our best performing NLP-radiomics pipeline (neural networks with the EN6 ROI) on the training and test sets. The results of the same radiomics model (neural networks with EN6 ROI) when trained and tested using expert-extracted pain labels, together with the results from a prior study by Wakabayashi et al. are provided for comparison. The reason for having high specificity and low sensitivity in our test set is explained in the discussion section

| **id** | **api** | **vdp** | **pain score** | **id** | **api** | **vdp** | **pain score** | **id** | **api** | **vdp** | **pain score** |
| --- | --- | --- | --- | --- | --- | --- | --- | --- | --- | --- | --- |
| p1 | -1 | no pain | none | p67 | 1 | pain | severe | p133 | 0.14 | pain | moderate |
| p2 | -0.5 | no pain | none | p68 | 1 | pain | severe | p134 | 1 | pain | moderate |
| p3 | -0.33 | no pain | none | p69 | 0.5 | pain | severe | p135 | 1 | pain | moderate |
| p4 | -0.33 | no pain | none | p70 | 0.5 | pain | severe | p136 | 0.82 | pain | moderate |
| p5 | -1 | no pain | none | p71 | 1 | pain | severe | p137 | 1 | pain | moderate |
| p6 | -1 | no pain | none | p72 | 0.43 | pain | severe | p138 | 1 | pain | moderate |
| p7 | -1 | no pain | none | p73 | 0.33 | pain | severe | p139 | 0.45 | pain | moderate |
| p8 | -0.5 | no pain | none | p74 | 1 | pain | severe | p140 | 1 | pain | moderate |
| p9 | -1 | no pain | none | p75 | 0.67 | pain | severe | p141 | 0.33 | pain | moderate |
| p10 | -0.33 | no pain | none | p76 | 0.2 | pain | none | p142 | 0.47 | pain | moderate |
| p11 | -1 | no pain | none | p77 | 1 | pain | none | p143 | 0.6 | pain | moderate |
| p12 | -0.5 | no pain | none | p78 | 0 | pain | none | p144 | 1 | pain | moderate |
| p13 | -1 | no pain | none | p79 | 0.6 | pain | none | p145 | 0.14 | pain | moderate |
| p14 | -1 | no pain | none | p80 | 0.5 | pain | none | p146 | 1 | pain | moderate |
| p15 | -1 | no pain | na | p81 | 0 | pain | none | p147 | 0 | pain | moderate |
| p16 | -1 | no pain | na | p82 | 1 | pain | na | p148 | 1 | pain | moderate |
| p17 | -1 | no pain | na | p83 | 1 | pain | na | p149 | 0.67 | pain | mild |
| p18 | -1 | no pain | na | p84 | 1 | pain | na | p150 | 0.6 | pain | mild |
| p19 | -0.14 | no pain | na | p85 | 1 | pain | na | p151 | 1 | pain | mild |
| p20 | -1 | no pain | moderate | p86 | 1 | pain | na | p152 | 1 | pain | mild |
| p21 | -0.43 | no pain | moderate | p87 | 0.5 | pain | moderate | p153 | 0 | pain | mild |
| p22 | -0.33 | no pain | mild | p88 | 0 | pain | moderate | p154 | 0.5 | pain | mild |
| p23 | -1 | no pain | mild | p89 | 0.87 | pain | moderate | p155 | 0.11 | pain | mild |
| p24 | -1 | no pain | mild | p90 | 0.5 | pain | moderate | p156 | 0.33 | pain | mild |
| p25 | 0.33 | pain | severe | p91 | 0.14 | pain | moderate | p157 | 0.5 | pain | mild |
| p26 | 1 | pain | severe | p92 | 1 | pain | moderate | p158 | 1 | pain | mild |
| p27 | 0.67 | pain | severe | p93 | 0.2 | pain | moderate | p159 | 0 | pain | mild |
| p28 | 0.33 | pain | severe | p94 | 1 | pain | moderate | p160 | 0.45 | pain | mild |
| p29 | 1 | pain | severe | p95 | 0.2 | pain | moderate | p161 | 1 | pain | mild |
| p30 | 1 | pain | severe | p96 | 0 | pain | moderate | p162 | 1 | pain | mild |
| p31 | 0.5 | pain | severe | p97 | 1 | pain | moderate | p163 | 0 | pain | mild |
| p32 | 1 | pain | severe | p98 | 1 | pain | moderate | p164 | 0 | pain | mild |
| p33 | 0.33 | pain | severe | p99 | 1 | pain | moderate | p165 | 0.33 | pain | mild |
| p34 | 0 | pain | severe | p100 | 0.6 | pain | moderate | p166 | 0.14 | pain | mild |
| p35 | 0.5 | pain | severe | p101 | 0.5 | pain | moderate | p167 | 1 | pain | mild |
| p36 | 0.67 | pain | severe | p102 | 0.2 | pain | moderate | p168 | 1 | pain | mild |
| p37 | 0.33 | pain | severe | p103 | 1 | pain | moderate | p169 | 0.33 | pain | mild |
| p38 | 0.71 | pain | severe | p104 | 1 | pain | moderate | p170 | 0.14 | pain | mild |
| p39 | 1 | pain | severe | p105 | 1 | pain | moderate | p171 | 0.33 | pain | mild |
| p40 | 0.75 | pain | severe | p106 | 1 | pain | moderate | p172 | 1 | pain | mild |
| p41 | 0.67 | pain | severe | p107 | 0.5 | pain | moderate | p173 | 1 | pain | mild |
| p42 | 1 | pain | severe | p108 | 1 | pain | moderate | p174 | 0.64 | pain | mild |
| p43 | 1 | pain | severe | p109 | 1 | pain | moderate | p175 | 1 | pain | mild |
| p44 | 0.5 | pain | severe | p110 | 0.33 | pain | moderate | p176 | 0.67 | pain | mild |
| p45 | 0.75 | pain | severe | p111 | 1 | pain | moderate | p177 |  | na | mild |
| p46 | 0.56 | pain | severe | p112 | 1 | pain | moderate | p178 |  | na | mild |
| p47 | 1 | pain | severe | p113 | 1 | pain | moderate | p179 |  | na | mild |
| p48 | 1 | pain | severe | p114 | 1 | pain | moderate | p180 |  | na | moderate |
| p49 | 0.11 | pain | severe | p115 | 0.78 | pain | moderate | p181 |  | na | moderate |
| p50 | 0.6 | pain | severe | p116 | 0 | pain | moderate | p182 |  | na | moderate |
| p51 | 0.6 | pain | severe | p117 | 1 | pain | moderate | p183 |  | na | moderate |
| p52 | 1 | pain | severe | p118 | 1 | pain | moderate | p184 |  | na | moderate |
| p53 | 0.67 | pain | severe | p119 | 0.5 | pain | moderate | p185 |  | na | moderate |
| p54 | 1 | pain | severe | p120 | 0.6 | pain | moderate | p186 |  | na | moderate |
| p55 | 1 | pain | severe | p121 | 1 | pain | moderate | p187 |  | na | na |
| p56 | 1 | pain | severe | p122 | 1 | pain | moderate | p188 |  | na | na |
| p57 | 1 | pain | severe | p123 | 0.5 | pain | moderate | p189 |  | na | na |
| p58 | 0.71 | pain | severe | p124 | 0.33 | pain | moderate | p190 |  | na | na |
| p59 | 0 | pain | severe | p125 | 0.56 | pain | moderate | p191 |  | na | na |
| p60 | 0 | pain | severe | p126 | 0.67 | pain | moderate | p192 |  | na | na |
| p61 | 0.33 | pain | severe | p127 | 1 | pain | moderate | p193 |  | na | na |
| p62 | 0 | pain | severe | p128 | 0.67 | pain | moderate | p194 |  | na | none |
| p63 | 0.33 | pain | severe | p129 | 1 | pain | moderate | p195 |  | na | severe |
| p64 | 1 | pain | severe | p130 | 0.33 | pain | moderate | p196 |  | na | severe |
| p65 | 0.2 | pain | severe | p131 | 0.33 | pain | moderate | p197 |  | na | severe |
| p66 | 0.43 | pain | severe | p132 | 1 | pain | moderate |  |  |  |  |

**Figure A2:** Relation between expert-extracted pain scores and NLP-extracted average pain intensities (API). The box plot is generated using the pyplot package in python.
